# Integrated Volatile Compounds and Transcriptional Gene Analysis Elucidate the Deterioration Mechanism of Embryo Rice During Storage

**DOI:** 10.3390/foods14091482

**Published:** 2025-04-24

**Authors:** Xiyuan Yang, Tingting Su, Lixue Ma, Xindi Mu, Hui Wang, Lei Xu, Lidong Wang, Baijun Wang, Di Yao, Changyuan Wang

**Affiliations:** 1 College of Food Science, Heilongjiang Bayi Agricultural University, Daqing 163319, China; 13555517359@163.com (X.Y.); sueting1126@163.com (T.S.); 15765973693@163.com (L.M.); mxd15845072658@163.com (X.M.); 13803901709@163.com (H.W.); xulei7701@163.com (L.X.); wanglidong-521@163.com (L.W.); byndwcy@163.com (C.W.); 2 National Coarse Cereals Engineering Research Center, Daqing 163319, China; 3 Heilongjiang Beidahuang Rice Industry Group Co., Ltd., Harbin 150000, China; 18745937651@163.com; 4 Key Laboratory of Agricultural Products Processing and Quality Safety of Heilongjiang Province, Daqing 163319, China

**Keywords:** embryo rice, storage, HS-SPME-GC-MS, transcriptome, quality deterioration

## Abstract

Embryo rice, as a product of processing rice, improves palatability and retains the nutritional characteristics of brown rice. However, the storage period of embryo rice is only 30 d at room temperature. To delay the deterioration in the quality of embryo rice during storage, this study used polyethylene terephthalate/aluminum foil/polyethylene (PET/AL/PE) to vacuum-package embryo rice, and analyzed the quality changes under 25 or 4 °C storage conditions. At the same time, volatile compound analysis and transcriptomic analysis were integrated to explore the quality deterioration mechanism of embryo rice during storage. The electronic nose results showed that the odor of embryo rice changed significantly during different storage periods (*p* < 0.05). A total of 72 volatile compounds were identified by Headspace–Solid-Phase Micro-Extraction–Gas Chromatography–Mass Spectrometry (HS-SPME-GC-MS), with 2-pentylfuran, naphthalene, and styrene contributing the most in the early stage, and 2-hexenal, nonanal, trans-2-nonenal, and ethanol contributing more in the later stage. Correlation analysis showed that fatty acids, malondialdehyde (MDA), lipase, and ferric-reducing antioxidant power (FRAP) were positively correlated with aldehydes and acids (*p* < 0.05), while catalase (CAT) and 2,2-diphenyl-1-picrylhydrazyl (DPPH) were negatively correlated (*p* < 0.05). This was mainly because the oxidative decomposition of lipids and the weakening of antioxidant capacity would lead to the accumulation of aldehydes. In the Mantel test analysis, color had the strongest correlation with volatile compounds, followed by taste value, and finally texture. In transcriptomic analysis, lipid synthesis and metabolism were key pathways for the storage deterioration of embryo rice, and the *LOX* gene played an important regulatory role. These results can provide a theoretical basis for the evaluation of quality and selection of storage method of embryo rice.

## 1. Introduction

Embryo rice refers to rice with an embryo retention rate of more than 80% [1], which combines the taste of white rice with the nutritional value of brown rice, and is a very ideal staple food [2] which is in great market demand. Due to the milling process, the germ loses the protection of the bran layer and is susceptible to oxygen, temperature, and humidity [3]. Therefore, embryo rice is prone to oxidative rancidity and quality deterioration during storage, but because of its strong spontaneous respiration, it will secrete a large amount of lipase, amylase, and protease, which promotes the metabolism of nutrients and aggravates quality deterioration [4]. At normal temperature, germ rice can only be stored for about 30 d, which makes it difficult to meet the needs of market production, processing, transportation, and sales [5]. Therefore, improving storage conditions and upgrading storage technology to protect storage quality and extend shelf life have become urgent problems for scholars domestically and internationally. Currently, research on the storage of embryo rice mainly focuses on nutritional [6], sensory [7], and quality indexes [8], but there are few studies on the changes in volatile components and the deterioration mechanism.

Volatile compounds play a crucial role in the formation of food flavor, and their types and contents are affected by many factors, including storage conditions and surrounding temperature [9]. During grain storage, the changes in volatile compounds are usually closely related to lipid oxidation and enzymatic reactions [10]. It has been shown that the storage process of rice will produce aldehydes, alcohols, ketones, and other volatile compounds, and the accumulation of these compounds may lead to undesirable flavors, such as aging or a rancid taste [11]. Although there have been some studies on the changes in volatile compounds during embryo rice storage, most of these studies focus on chemical analysis and lack a genetic analysis of the deterioration in quality of embryo rice. The synthesis of volatile compounds involves complex metabolic networks, and their regulatory mechanisms may be affected by the expression of multiple genes [12]. Therefore, it is difficult to fully reveal the molecular mechanism of volatile compound changes through chemical analysis alone.

Transcriptome analysis, as a high-throughput technique, can fully reveal the changes in gene expression and provide a powerful tool for studying the regulatory mechanisms of metabolic pathways [13]. In recent years, the application of transcriptome analysis in the food science field has been increasing, especially in the study of the molecular mechanisms of quality change during food storage [14]. For example, through transcriptome analysis, researchers can identify key genes involved in lipid oxidation [15], antioxidant defense systems [16], and secondary metabolism [17], thereby revealing the molecular basis of food quality changes [18]. However, transcriptome studies on the changes in volatile compounds during the storage of embryo rice are still rare [19]. By combining the chemical analysis of volatile compounds with transcriptome data, the molecular basis of quality change during the storage of embryo rice can be more comprehensively revealed.

In this study, volatile compound changes were combined with transcriptomic analysis to systematically investigate the dynamic changes in volatile compounds and their gene regulation mechanisms during embryo rice storage. This combined strategy not only provided a new perspective for the in-depth understanding of the changes in volatile compounds in the storage process of embryo rice but also provided a solid scientific basis for developing targeted preservation technology of embryo rice and optimized storage process parameters by revealing key regulatory gene networks, which has important theoretical value and practical significance for improving the quality value of embryo rice and extending the shelf life.

## 2. Materials and Methods

### 2.1. Materials and Instruments

Longjing 91 was selected as the variety of embryo rice, and the rate of embryo retention was 90%. Embryo rice was provided by Beidahuang Rice Industry, Harbin, Heilongjiang in 2023.

The following reagents were used: hydrochloric acid standard titration solution, Xiamen Shengbiao Technology Co., Ltd., (Xiamen, China); sodium sulfite anhydrous, potassium sulfate, sodium hydroxide, peroxyacetic acid, bromocresol green, ethanol absolute, and concentrated sulfuric acid, Liaoning Quan Rui Reagent Co., Ltd., (Jinzhou, China); and boric acid, Tianjin Damao chemical partnership enterprise, (Tianjin, China).

### 2.2. Embryo Rice Storage Test

An amount of 100 g of embryo rice was vacuum-packed in 30 bags with PET-ALPE and stored at 25 or 4 °C with 50% relative humidity. Samples were collected every 60 d until 300 d.

### 2.3. Determination of Basic Indexes

#### 2.3.1. Physicochemical Index

The moisture content of the embryo rice was determined using the direct drying method in GB/T5009.3-2016 [20]. The protein content of embryo rice was determined using the Kjeldahl nitrogen determination method in GB/T 5009.5-2016 [21]. The multiplication coefficient of nitrogen content to protein content was 5.95.

#### 2.3.2. Fatty Acid Value, Lipase Activity, and MDA Determination

The fatty acid value was determined by the method in GB/T 20569-2006 “Rules for Determining Storage Quality of Rice” [22]. Lipase was determined according to the method in GB/T 5523-2008 “Determination of Lipase activity of grain and oil” [23]. Malondialdehyde was tested according to the instructions of the malondialdehyde kit (No. G0109W, Suzhou Gris Biological Co., Ltd., Suzhou, China).

#### 2.3.3. Catalase, DPPH, and FRAP

Catalase was tested according to the instructions of the catalase kit (No. G0105W, Suzhou Gris Biological Co., Ltd.). DPPH was tested according to the instructions of the DPPH free radical scavenging ability kit (No. A153-1-1, Nanjing Jiancheng Co., Ltd., Nanjing, China). FRAP was tested according to the instructions of the Total Antioxidant Capacity (T-AOC) test kit specification (No. BC1310, Solebault life science, Beijing, China).

#### 2.3.4. Texture

Embryo rice (50 g), after rinsing and removing impurities, was mixed with 100 mL of distilled water, which was steamed in a rice cooker for 30 min. Then, it was kept warm for 30 min. The texture analyzer (TMS-PRO, Federal Trade Commission, MA, USA) was utilized to determine the texture properties in triplicate. The following parameter settings were used: disk probe, pre-test speed of 30 mm/min, test speed and post-test speed of 60 mm/min, initial force of 0.1 N, and deformation amount of 30%.

#### 2.3.5. Taste Value

The taste value of steamed embryo rice was measured using a rice taste meter (STA1B, Satake, Hiroshima-ken, Japan). The rice was cleaned for 30 s, soaked in a rice-to-water ratio of 1:1.35 for 30 min, and then placed in an iron can. The steamed embryo rice was blown 20 min, 8.0 g of which was placed into a stainless steel ring, which was pressed on a tablet press for 10 s. The pressed embryo rice cake was put into a rice flavor meter to measure the gloss, texture, completeness, taste, and flavor of the rice. According to the five indicators, the comprehensive score of the taste value was obtained.

#### 2.3.6. Color

The color of embryo rice was determined by color testers (CS-580, Hangzhou Caipu Technology, Hangzhou, China), and black and white calibration was performed before inspection. The L*, a*, and b* values of embryo rice were determined. The L* value represents the brightness of embryo rice; positive and negative a* values indicate the degree of red and green, respectively; and positive and negative values of b* indicate the degree of yellow and blue, respectively. ∆*E* represents the total color difference, indicating the difference in color of the embryo rice with storage time.

$$\Delta E=\sqrt{{∆L}^{2}+{∆a}^{2}+{∆b}^{2}}$$

### 2.4. Determination of Volatile Compounds

#### 2.4.1. E-Nose

The e-nose (C-nose, Shanghai Baosheng Technology, Shanghai, China) system consists of 28 metal oxide sensors. The cooked embryo rice (2.00 g) was placed in a headspace sample bottle (20 mL). An electronic nose probe was inserted to absorb overhead air and determine the volatiles. The following setup parameters for the e-nose were set: cleaning time of 120 s, sensor cleaning flow rate of 6 L/min, determination time of 60 s, injection flow rate of 1 L/min, and phase of signal acquisition of 55–60 s. Three replicate measurements were taken for each sample.

#### 2.4.2. HS-SPME-GC-MS

The study made a slight modification to the method of Sehun Choi [24]. The cooked embryo rice (2.00 g) was transferred into a 20 mL (22.5 × 75 mm) glass headspace vial (Gerstel, Baltimore, MD, USA). The headspace bottle was placed in a constant-temperature heating device and maintained at 40 °C for 5 min to achieve a distribution balance between the sample matrix and the headspace phase. Then, the solid-phase micro-extraction fiber head (50/30 μm DVB/CAR/PDMS, Supalco, PA, USA) was inserted and extracted at 60 °C for 40 min to adsorb the target volatile compounds in the overhead space. An ISQ series single-quadrupole (Thermo Fisher Scientific, Waltham, MA, USA) column gas chromatograph equipped with a DB-Wax capillary column (30 mm × 0.25 mm i.d., 0.25 μm film thickness; Agilent Technologies, Santa Clara, CA, USA) was used to separate the components at 40 °C for 5 min, followed by a gradient of 5 °C/min to 230 °C to ensure effective separation of the components. The temperature was held at 230 °C for 10 min, the solvent delay was set to 3 min, the inlet temperature was kept constant at 260 °C, helium with 99.999% purity was selected as the mobile phase, the flow rate was controlled to 1.0 mL/min, and the detection sensitivity was improved by adopting the non-shallow-flow injection mode. When the interface temperature was 260 °C, the ion source (EI) temperature was 200 °C, and the electron bombardment energy was 70 eV, scanning was carried out in the range of *m*/*z* 35.0–600.0, and the solvent delay time was 3 min to eliminate column loss interference.

Qualitative analysis: After mass spectrometry deconvolution of GC-MS spectra, compounds with a matching degree greater than 75 were selected for analysis and compared with the NIST database. Under the same chromatographic conditions, the retention index of the target was calculated and compared with the NIST database RI to identify the target.

Quantitative analysis: The relative content of each compound was calculated according to the peak area normalization method of each volatile compound.

#### 2.4.3. Evaluation of Major Volatile Compounds

The compounds with VIP value >1 were screened by OPLS-DA, and then, the main volatile components of the storage stage were determined. According to the method of Liu [25], the ROAV of major volatile compounds of embryo rice during storage were calculated to evaluate the impact of specific components on the whole flavor of rice during storage. The ROAV value of compounds that has the greatest effect on the whole flavor of the sample was defined as 100, that is, ROAVmax = 100, while that of the other groups were

$$ROAVx=100\times \frac{OAVx}{OAVmax}\approx 100\times \frac{Cx}{Tx}\times \frac{Tmax}{Cmax}$$

where Cx is the proportional content of each volatile compound, %; Tx is the sensory threshold corresponding to each volatile compound, mg/kg; Cmax is the proportional content of the ingredient which exerts the greatest influence on the whole flavor within the sample, %; and Tmax is the sensory threshold corresponding to the component within the sample that exerted the greatest influence on the whole flavor, mg/kg.

### 2.5. Transcriptome Analysis

#### 2.5.1. RNA Extraction, Library Construction, and Sequencing

Samples of embryo rice in different storage periods were taken, and TRIzol^®^ reagent was used to extract total RNA from rice tissues in accordance with the instructions. DNase I was used to remove genomic DNA. The concentration and purity of the RNA were determined by Nanodrop2000, and the integrity of the RNA was detected by 1% agarose gel electrophoresis. High-quality RNA samples (OD260/280 ≥ 1.8; OD260/230 ≥ 1.0; 23S:16S ≥ 1.0; concentration ≥ 50 ng/L; total amount ≥ 1 μg) were screened for subsequent library construction. After enrichment, Phusion DNA polymerase was used for PCR amplification, and 15 cycles were amplified. After quantification of TBS380, Illumina Novaseq was used for RNA-seq double-ended sequencing. The data generated by the Illumina platform were used for bioinformatics analysis. All analyses were carried out using the cloud platform of Shanghai Majorbio Bio-pharm Technology Co., Ltd. (Shanghai, China).

#### 2.5.2. Differential Expression Analysis and Functional Enrichment

Based on the Burrows–Wheeler method, the quality sequence obtained after quality control was compared with the specified reference genome to obtain the characteristic sequence information of the sequenced sample. RSEM (http://deweylab.github.io/RSEM/) (accessed on 1 January 2025) was used for single- or double-side sequencing data for gene expression analysis. The difference analysis software DESeq2 version 3.21 (http://bioconductor.org/packages/stats/bioc/DESeq2/) (accessed on 12 January 2025) with a negative binomial distribution was used to analyze the differential expression of genes between samples. The sample genes were annotated and classified according to the GO database (Gene Ontology, http://www.geneontology.org/) and KEGG database (Kyoto Encyclopedia of Genes and Genomes, http://www.genome.jp/kegg/) (accessed on 24 January 2025), and the differential genes were enriched using GO and KEGG analysis.

### 2.6. Data Analysis

Microsoft Excel 2019 and SPSS 20.0 were used for the data processing. Image processing was carried out using Origin 2024 and Chiplot. Univariate analysis of variance and the *t*-test were used to analyze the significant difference, and the significance level of all tests was *p* < 0.05.

## 3. Results and Discussion

### 3.1. Changes in Basic Indexes During Embryo Rice Storage

#### 3.1.1. Changes in Oxidation Index During Embryo Rice Storage

**Figure 1:**
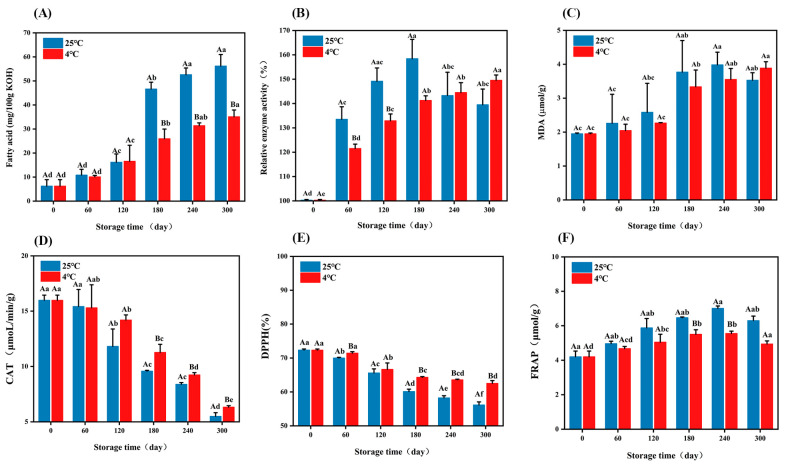
Changes in (**A**) fatty acid, (**B**) lipase, (**C**) malondialdehyde, (**D**) catalase, (**E**) DPPH, and (**F**) FRAP during embryo rice storage. The values represent the mean ± standard deviation. Capital letters indicate significant differences at two temperatures (*p* < 0.05), and lowercase letters indicate significant differences at storage times of 0–300 d (*p* < 0.05).

Fatty acid is the hydrolyzed product of fat, which is an important index that reflects the quality of embryo rice. The higher the fatty acid content, the worse the quality of rice [26]. Fatty acid values gradually increased with the extension in storage time (Figure 1A). At the two temperatures, the fatty acid value increased from 6.23 to 56.19 and 35.10 mg/100 g KOH, respectively. The fatty acid value at 25 °C increased faster than that at 4 °C. In this study, the fatty acid value (35 mg/100 g KOH) of the sample was set as the limit of shelf life, beyond which it was considered to be stale [27]. Compared with the results of Xu et al. [28], the shelf life of embryo rice was extended at both storage temperatures. The shelf life at 25 °C was 120 d, which was 240 d at 4 °C. The increase in fatty acid value was closely related to the lipase activity of the embryo rice [29]. The relative enzyme activity first increased and then decreased, and was relatively slow at 4 °C (Figure 1B), which was consistent with the results of Li et al. [30]. Lipase activity increased first and then decreased during storage, mainly due to the activation of endogenous enzymes and the increase in exogenous enzymes produced by microorganisms in the initial stage of storage, and decreased later due to the consumption of substrate fat. At 25 °C, lipase activity reached a maximum of 158.41% in 180 d and 149.56% in 300 d at 4 °C. MDA first increased and then decreased (Figure 1C). The increase in MDA content is mainly due to the fact that fatty acids can be produced during the storage process of embryo rice, which degrade to form some small molecules such as MDA, while the subsequent decrease in MDA is due to its volatility and can be released into the air in the form of gas [31]. At the same storage time, the MDA content of embryo rice at 25 °C was higher than that at 4 °C. The results indicated that high temperature accelerated the process of fat oxidation and MDA was one of the products of fat oxidation.

The catalase decreased continuously during storage (Figure 1D), mainly because, during the storage process, rice will breathe and consume nutrients, the ability of CAT synthesis and renewal will decrease, and the decomposition process of enzymes will accelerate, resulting in a decrease in the overall content and activity of CAT [32]. With an extension in storage time, DPPH decreased (Figure 1E). This may be because polyphenols in the germ are the main antioxidant components, and with the extension of storage time, fewer polyphenols are retained in the germ [33]. The FRAP value increased first and then decreased with storage time (Figure 1F). The early increase in FRAP may be due to the gradual release or transformation of some potential antioxidant substances in embryo rice in the early storage stage. The late decline in FRAP was mainly due to the gradual decomposition of antioxidant substances during storage, enzymatic hydrolysis, or microbial action, which led to a decrease in reducing capacity.

#### 3.1.2. Changes in Sensory Quality of Embryo Rice During Storage

**Figure 2:**
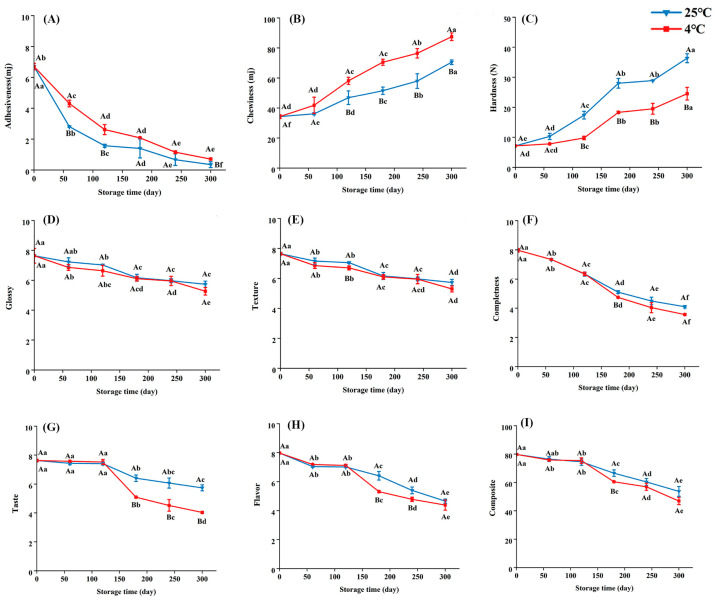
Changes in texture and taste value during embryo rice storage. (**A**) Adhesiveness, (**B**) chewiness, (**C**) hardness, (**D**) gloss, (**E**) texture, (**F**) completeness, (**G**) taste, (**H**) flavor, and (**I**) composite. The values represent the mean ± standard deviation. Capital letters indicate significant differences at two temperatures (*p* < 0.05), while lowercase letters indicate significant differences at storage times of 0–300 d (*p* < 0.05).

Figure 2A–C show the changes in texture in embryo rice at different storage times. The texture of rice is an important indicator reflecting the edible quality of rice, including springiness, chewiness, and hardness [34]. Figure 2A shows the adhesiveness reflecting the size of the binding force between rice molecules. The weakening of the gathering ability of the rice leads to a loose texture, reduced adhesion, and poor taste [35]. Figure 2B shows chewiness, which represents the energy required to chew and swallow an elastic sample [36]. Chewiness increased with storage time, and the lower the moisture, the greater the chewiness. The moisture decreased with the increase in storage time (Figure S1A). Figure 2C shows that the hardness increased after 300 d of storage, and the growth rate of 25 °C was 9.5% faster than that at 4 °C, mainly because of the decline in crude protein during storage, which represents proteolytic denaturation, and its network structure is more easily attached, resulting in increased hardness (Figure S1B) [8].

The taste value is a crucial indicator of the quality of embryo rice during storage. In this study, this value decreased with storage time. Initially, it exhibited a gloss of 7.64, texture of 7.65, completeness of 7.97, taste of 7.62, flavor of 7.98, and composite of 79.75. Figure 2D–F show that gloss declined more slowly than the other indexes, and texture, completeness, and flavor all showed a clearer change trend within 300 d of storage. Taste showed a significant difference between the two temperatures, indicating that temperatures strongly affected flavor (Figure 2G). Figure 2I shows that the difference in composite taste was not significant at the two temperatures, and 4 °C can be chosen for short-term consumption, while storage at 25 °C was recommended more for long-term storage. The main reason is that during short-term storage, low temperatures are more likely to protect the taste and aroma of the embryo rice. Long-term storage can damage the internal structure of the embryo rice [37].

When choosing rice for consumption, color provides the consumer with a first impression of the product [38]. The L* and a* values decreased and the b* value increased during storage (Table 1). These results were similar to the conclusion of Yang et al. [39] in terms of the change in L* value, but there were differences in the magnitude of decline. At 25 °C, L* and a* decreased 11.86 and 80%, respectively, and b* increased 27.79%. At 4 °C, L* and a* decreased 23.99 and 70.27%, respectively, and b* increased 18.03%. The decrease in L* is due to the formation of colored substances on the surface of rice, while the increase in b* may be related to lipid oxidation and the Maillard reaction. The Δ*E* value at the two temperatures changed with the increase in storage time, and the larger the Δ*E* value, the greater the change with storage time [39]. The change in Δ*E* at 25 °C was relatively gentle, while the change in Δ*E* at 4 °C was more significant (Table 1). The difference between the two temperatures was not significant in the early stage, and the change became significant after 240 d. In terms of color, it is recommended to choose 4 °C for short-term storage and 25 °C for long-term storage. Long-term low-temperature refrigeration may cause rice grains to turn yellow-brown.

### 3.2. Analysis of Volatile Compounds of Embryo Rice During Storage

#### 3.2.1. Analysis of Odor of Embryo Rice During Storage

**Figure 3:**
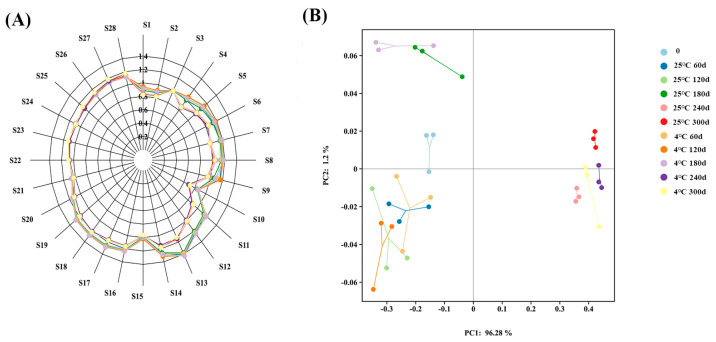
(**A**) Radar map and (**B**) PCA of embryo rice at two temperatures.

The e-nose system can detect and analyze subtle changes in the taste and aroma of different samples in real-time and objectively, mimicking the human sense of smell [40]. The results of the 28 e-nose sensors can be found in Table S1. There are significant differences in S11, S12, and S13, indicating a reduction in ketones, alcohols, and methane compounds (Table S1, Figure 3A). The difference between S6 and S7 is significant, indicating a decrease in aromatic compounds and alkanes. In addition, the significant change at 4 °C is larger than that at 25 °C (Figure 3A). This indicates that the low-temperature environment will affect the flavor and aroma of the embryo rice, especially aldehydes, alcohols, and aromatic compounds.

The sum of PCA1 and PCA2 was 97.48%, indicating that the PCA outcomes were generally precise and could be employed to analyze the odor data of all samples (Figure 3B). PCA1 contributed 96.28% of the total variance, while PCA2 contributed 1.2%. The samples were categorized into eleven different groups based on the PCA formula. The results indicated that the e-nose sensor can successfully distinguish embryo rice with different storage periods. The flavor separation of embryo rice within 240–300 d compared to that at 0 d was clear, and the deterioration was more substantial in the later period. The main reasons are protein degradation and fat oxidation, which lead to the deterioration in embryo rice and its aroma.

#### 3.2.2. Analysis of HS-SPME-GC-MS of Embryo Rice During Storage

**Figure 4:**
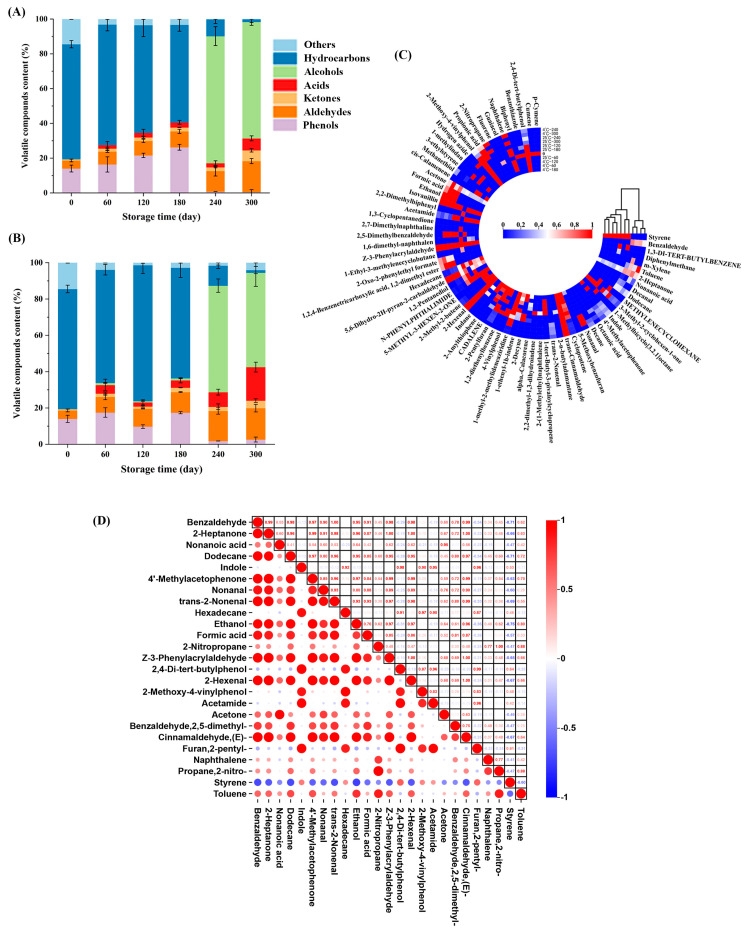
Analysis of volatile compounds during embryo rice storage: relative content at (**A**) 25 and (**B**) 4 °C; (**C**) heat maps; and (**D**) ROAV correlation analysis.

The key characteristic that defines the aroma of food after storage is volatility, which is a key point in determining whether the food is spoiled [41]. Spoiled food produces unpleasant odors that can have a significant impact on consumer preferences. To assess the alterations in embryo rice stored at two different temperatures over a period of 300 d, HS-SPME-GC-MS was employed to analyze the key volatile compounds present in the samples. The distribution of the content of volatile compounds in embryo rice during storage is shown in Figure 4A,B. The relative content of hydrocarbons was the highest before 180 d, the aldehydes showed an increasing trend, and alcohol compounds increased significantly overall at 240–300 d, which indicated that the types and contents of volatile compounds varied greatly after storage for 180 d. The fluctuation trend at different temperatures was clear, indicating that both temperature and storage time can affect the stability of the compounds.

A total of 72 volatile compounds were categorized for the embryo rice samples during storage (Table S2). With reference to the relative content of each volatile compound, heat maps were drawn to visualize the volatile composition of the embryo rice at both temperatures more lucidly and straightforwardly (Figure 4C). A total of 10 aldehydes were found in this study, and their relative contents increased with the prolongation of storage time. There were three types of aldehydes at 0 d. Z-3-Phenyltyralaldehyde is a fragrance with a spicy cinnamon aroma. Trans-cinnamaldehyde has a sweet, spice, candy aroma [42]. 2-Hexena has a sweet almond flavor. The relative content of three aldehydes increased the most during storage, but also increased the most at 4 °C. 5,6-Dihydro-2H-pyran-2-carbaldehyde and isovanillin appeared at two temperatures on day 60. 5,6-Dihydro-2H-pyran-2-carbaldehyde has an aldehyde aroma. Isovanillin has an olive-like aroma and a strong milky aroma, which can vary with changes in ambient temperature. Benzaldehyde, nonanal, and trans-2-nonenal appeared in late storage. Benzaldehyde is mainly derived from linoleic acid and rises continuously during storage [43]. Nonanal has a cured meat aroma and trans-2-nonenal has a fat aroma. The appearance of the three in the later stage indicated that the odor of the embryo rice had deteriorated. 2,5-dimethylbenzaldehyde and decanal are present only at 25 °C, indicating that higher temperatures produce more aldehydes.

Hydrocarbons are the main volatile components in embryo rice storage, with the highest variety and content. There are 42 types of hydrocarbons during storage, among which there are 6 types of alkanes, most of which do not produce an aroma and have a limited contribution to the whole aroma of embryo rice. Although 1-methylbicyclo [3.2.1] octane, 2-n-butyladamantane, and 1-ethyl-3-methylenecyclobutane appeared at 0 d, it contributed little to the aroma of embryo rice during storage. Barring alkanes, most olefins play a negligible role in shaping the overall aroma [44]. During storage, 11 olefins appeared, and cyclo-pentene had a foul odor at 25 °C, which can easily cause deterioration and produce unpleasant odors. Styrene appeared at two temperatures for 0–180 d and has a fragrant floral aroma, so it decreased. Alpha-calacorene appeared at two temperatures in earlier storage, with a woody odor. 5-methyl-3-hexen-2-one has a sweet, berry, cheese aroma that appeared at 4 °C for 60–120 d. Within 300 d of storage, a total of 24 aromatic hydrocarbon compounds were detected. In fact, aromatic compounds were the main volatile compounds in the rice, but many compounds only appeared within 0 d, such as biphenyl (pleasant odor), fluorene (fruity flavor), and p-cymene (mild, pleasant, citrus). 2-Pentylfian (fruit flavor) is retained for the longest time and gradually fades with storage time [45]. The primary oxidation products impart a sweet or fruity aroma to rice during the oxidation process, but excessive production can also cause lipid oxidation and produce a rancid taste [46]. The presence of naphthalene in the storage process indicates that the embryo rice produces a tar taste. Toluene has a fragrant aroma similar to benzene and only appears in 240–300 d. Excessive toluene is toxic. It can be observed that aromatic hydrocarbons are less easily preserved and evaporate faster at 25 °C. Temperature can affect the retention time of aromatic hydrocarbons [47]. Alkynes are unsaturated derivatives of alkanes and olefins, with two types detected. 2-Decyne exhibits a pungent odor during the later stages of storage.

The volatile compounds in embryo rice during storage are rich in phenols and ketones, with 3 phenols and 5 ketones detected. Phenolic compounds can form a rice bran odor during the storage of embryo rice, affecting the flavor of the rice [48]. 2,4-Di-tert-butylphenol (phenolic flavor, stone carbon sour taste) and 2-methoxy-4-vinylphenol (sweet and spicy fruity aroma) appeared within 0–300 d during storage. Guaiacol (phenolic odor) only appeared in 300 d at 4 °C. Guaiacol and 2-methoxy-4-vinylphenol indirectly reflect the deterioration in embryo rice, forming a rice bran odor that affects the quality of food consumption. Research has found that volatile ketone compounds have a slightly fruity or sour–sweet taste. Acetone (mint scent) and 2-heptanone (fruity, spicy) appeared at 0 d, and acetone is a representative compound of aliphatic ketones. 3-Methyl-2-cycloexen-1-one (caramel odor), 4-methyletophenone (acetophenone odor), and the 2-oxo-2-phenylethyl format appeared in the later stages of storage, with their contents continuously increasing.

In addition to the volatile compounds mentioned above, embryo rice also contains acid and alcohol compounds which contribute to the complex aroma characteristics of embryo rice during storage. Acids are produced through lipid hydrolysis, and acetamide appears on day 60 with a rat odor. Most of the acid compounds appeared during late storage, which reflected the rancidity of the rice. However, hydrogen acid is limited by temperature and only appears at 25 °C. Alcohols are regarded as secondary products resulting from the oxidation of unsaturated fatty acids. These alcohols are formed through the further decomposition of aldehydes [49]. With the extension in storage time for embryo rice, 1,2-pentanediol and ethanol appeared [50].

#### 3.2.3. Analysis of ROAV of Embryo Rice During Storage

As shown in Figure S2A, a clustering of 11 groups of volatile compounds can be observed. The group at 0 d was dissociated from other groups. The separation between the rice stored for 60–180 d and the embryo rice stored for 240–300 d at two temperatures was clear. The results indicated that the changes in the embryo rice during the late storage period were clear. Figure S2B shows the screening of VIP > 1 volatile compounds during the storage of embryo rice. A total of 45 volatile compounds with significant contributions were selected and combined with the PCA plot in Figure S2A to obtain the hyperbolic plot in Figure S2C. The first group at 0 d is mainly located in the lower right quadrant, such as 1-ethenyl-1h-indend, N-phenylphthalimide, and 3-ethylstyrene. The second group at 60–180 d is at both temperatures and is mainly located in the upper right quadrant, such as naphthalene, alpha-calacorene, and 2-Decyne. Two groups mainly revolve around hydrocarbons. The last group is the 240 to 300 d group, located in the upper and lower left quadrant, which may be some ketones and aldehydes, such as acetone and Z-3-phenylacrylaldehyde.

The aroma of a food sample cannot be simply accounted for by the content of a single compound. The proportional content of volatile odor compounds cannot directly indicate the impact of compounds on the overall odor of the sample. People’s olfactory sensitivity to different compounds is very different, so it is necessary to combine the compound content with its odor threshold to evaluate the contribution of compounds to the overall odor [51]. Commonly, components that possess an ROAV equal to or higher than 1 are seen as the principal flavor compounds of the sample. By contrast, components with an ROAV falling within the range of 0.1 to 1 (not including 1) have significant impacts on adjusting the overall flavor of the sample [52]. According to VIP > 1 (Figure S2B), 45 main volatile compounds were screened. The threshold was determined by consulting the data, where the compounds for which the threshold cannot be determined were not analyzed. Table S3 reveals the ROAV values for 25 compounds. For ROAVs of aldehydes and hydrocarbons >1, the greatest contribution was made during the storage of embryo rice. Aldehydes have the largest ROAV values, possibly because aldehydes are relatively abundant during storage and the threshold is low. Thus, this will cause the ROAV to be over 100. 2-Pentylfuran, naphthalene, and styrene contribute most in the early stage. 2-Hexenal, nonanal, trans-2-nonenal, and ethanol contributed more in the later stage. The temperature of 25 °C had the greatest effect on most of the compounds, indicating that the higher the temperature, the more volatile the compounds are, resulting in a poor quality of the rice. A total of 25 compounds with an ROAV value mentioned above were selected for pairwise relevance analysis. As shown in Figure 4D, red and blue represent positive and negative correlations, respectively. Most volatile compounds were positively correlated, except for very few hydrocarbons. There was a significant positive correlation between aldehydes and acids. Styrene showed a significant negative correlation with other volatile compounds. Because styrene smells like balsamic flowers, the smell decreases with storage time. The change in 2-pentylfuran (fruity) was consistent with that of styrene. Indole has a fecal odor that appears in late storage and is negatively correlated with other aromatic volatile compounds. Thus, the production of one substance will affect the content of another substance in embryo rice during storage, which indicates that HS-SPME-GC-MS is a feasible and reliable method for detecting certain volatile compounds in embryo rice and determining the aroma quality of embryo rice during storage [53]. The interaction of the compounds had a certain impact on the aroma quality of embryo rice during storage.

#### 3.2.4. Correlation Analysis Between Volatile Compounds and Basic Indexes

**Figure 5:**
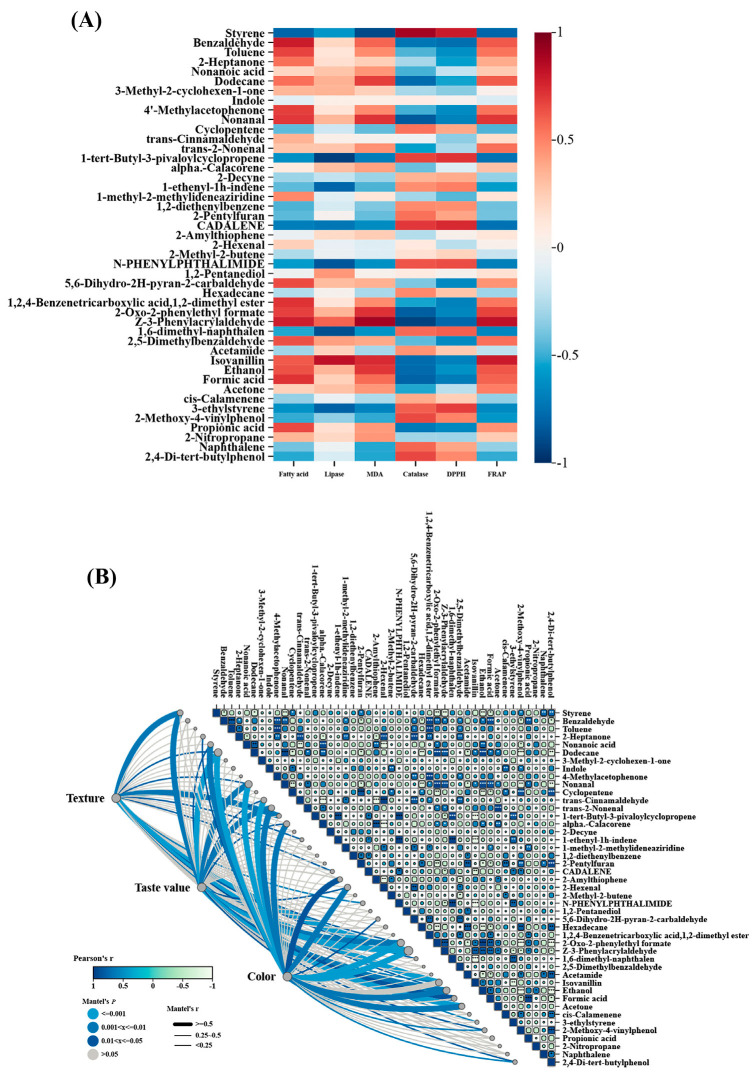
Main volatile compounds. (**A**) Correlation analysis and (**B**) Mantel test analysis of embryo rice during storage. Note: * is *p* < 0.5, ** is *p* < 0.001, *** is *p* < 0.0001.

Figure 5A shows the correlation analysis of lipid metabolism and oxidation with volatile compounds. The volatile compounds were positively correlated with fatty acids, malondialdehyde, lipase, and FRAP, and negatively correlated with catalase and DPPH, especially aldehydes and acids. In the storage of embryo rice, acids can create an environment conducive to the action of lipase, accelerate the hydrolysis of fat and increase fatty acids, and also participate in the oxidation reaction to increase malondialdehyde. Aldehydes cause fat oxidation, which not only generates more fatty acids and malondialdehyde, but also promotes more lipase synthesis, so acids and aldehydes are positively correlated with the changes in the three. Acids can promote the formation of antioxidant substances to increase the FRAP value. Although aldehydes often promote oxidation, special aldehydes also have antioxidant properties, so they are positively correlated with FRAP. The accumulation of acids and aldehydes may inhibit the activity of CAT, consume free radicals themselves, and reduce DPPH free radicals, so there is a negative correlation with the changes in CAT and DPPH in the storage of embryo rice.

Figure 5B shows the correlation between texture, taste value, and color and the main volatile compounds analyzed by the Mantel test. A thicker line represents a larger R-value, which indicates a stronger correlation between two matrices. The lighter the color, the smaller the *p*-value, indicating a more significant correlation. In general, the compounds that were most correlated with texture, taste value, and color difference in MT analysis were classified as hydrocarbons, ketones, and aldehydes. The influence of volatile compounds on the color difference is strongest, followed by texture and taste value. The main reason for the decline in texture is that with the extension in storage time, the relative content of hydrocarbons decreases, which leads to changes in hardness and viscosity. The reaction of ketones and aldehydes with protein, starch, and other components affected the retention and distribution of water and made the texture of the rice loose. The reaction of hydrocarbons, ketones, and aldehydes with the aroma components in the rice reduces or removes the original aroma substances, reduces the aroma quality, and leads to the decline in taste value. The Browning reaction and oxidation of volatile compounds during storage resulted in a decrease in the color difference of the remaining embryo rice.

### 3.3. Analysis of RNA-Seq of Embryo Rice During Storage

#### 3.3.1. Identification of DEGs Under Different Storage Conditions of Embryo Rice

**Figure 6:**
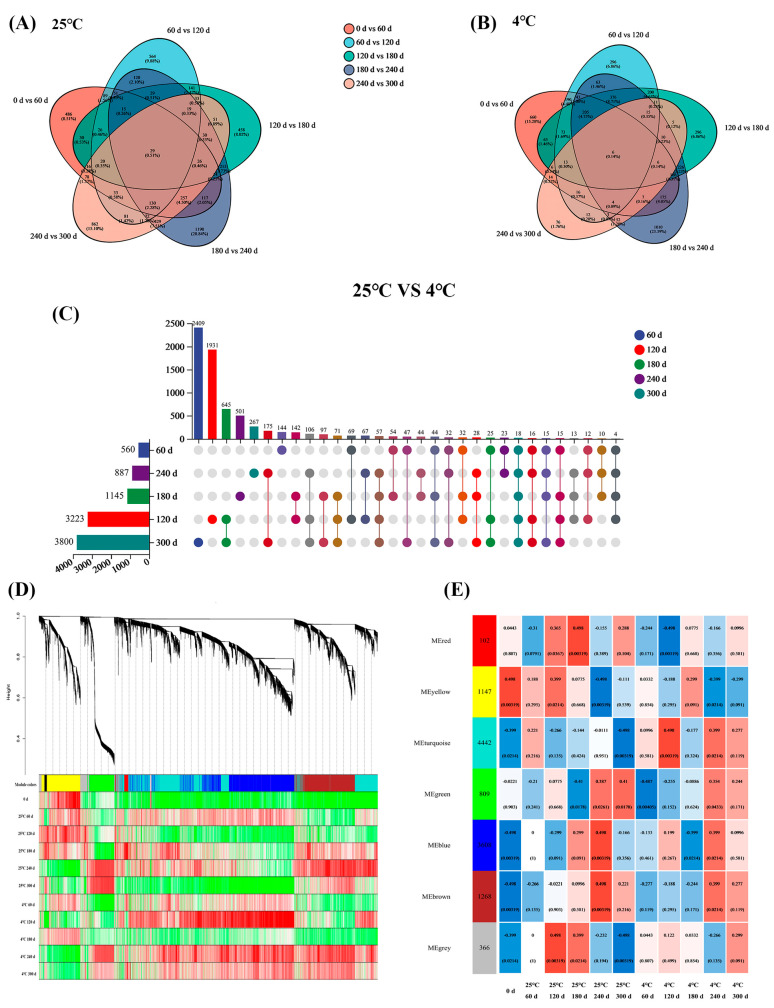
RNA-seq analysis of embryo rice stored at two temperatures for 300 d. The number of differentially expressed genes (DEGs) in the chart at (**A**) 25 and (**B**) 4 °C. (**C**) A map of DEGs of two temperatures at the same time. (**D**) Nine modules, each associated with a different color, formed from the major tree branches. (**E**) The association between modules and samples.

Figure 6A,B show the differentially expressed genes of the rice stored at 25 and 4 °C for 300 d, respectively. There were 29 common expression differences at 25 °C, and 6 at 4 °C. At both temperatures, the most different genes were found in the 180–240 d stage, possibly because embryo rice began to deteriorate in this stage. Figure 6C shows the number of different genes at the two temperatures at the same time. The horizontal bar chart on the left represents the statistical values of the elements of each set; in the middle matrix, a single point represents the unique elements of a certain set; the lines between points represent the unique intersections of different sets; and the vertical bar chart represents the corresponding intersections [45].

The number of different genes in the two groups at 60 d was closed, which may be mainly based on the adjustment of basic metabolism. At 300 d, the gene changes in the 25 °C group were significantly expanded. The results indicated that both temperature and storage time had an influence on the gene expression of embryo rice.

In order to explore the differences in gene expression patterns among embryo rice samples for different storage periods, correlation analysis was used to reveal the potential relationship between samples (Figure 6E). Color indicates the size of the correlation coefficient R^2^, and red is close to 1, indicating a strong correlation. The blue coefficient value is low and the correlation is weak. On the whole, the correlation between most samples was high, and most areas were red to light red, indicating that there was a strong internal correlation between the embryo rice samples. In the correlation analysis at two temperatures, R^2^ was 0.989 at 60 d of storage and 0.981 at 300 d of storage, mainly because temperature had little influence on the gene expression pattern and similar transcription products under the same packaging conditions. Among some samples with different numbers, for example, the R^2^ of the samples stored at 25 °C for 180 and 300 d was 0.961, and the correlation was between 0.9 and 0.96 at a medium level, indicating that with the increase in storage time, there were differences in gene expression patterns among samples, but there were still similar gene expression characteristics. When stored at 25 °C for 240 d, R^2^ was 0.772 compared with 0 d, and the correlation between samples was low, indicating that they had large differences in gene expression, which may be due to the different transcription conditions caused by storage time.

#### 3.3.2. Changes in Gene Expression Related to Lipid Metabolism

**Figure 7:**
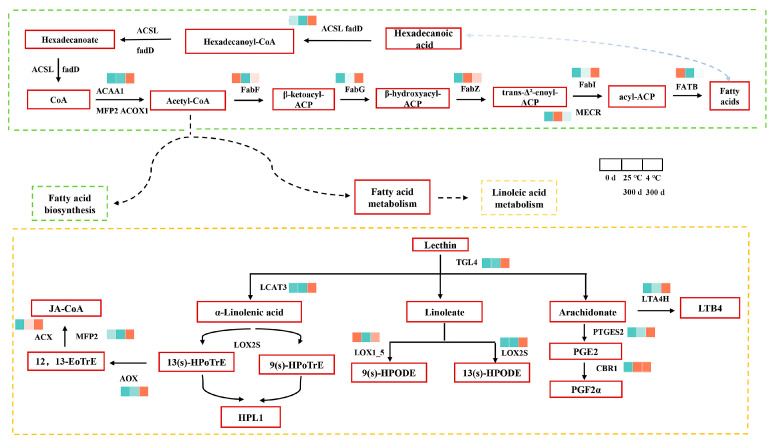
DEGs associated with lipid metabolism pathways enriched in KEGG pathways.

Through the accumulation of data in the early stage of the experiment, it can be found that lipid has a strong influence on the deterioration in rice during storage, and most of the formed volatile compounds are also caused by fatty acid changes [54]. Subsequently, lipid formation and metabolism were mainly analyzed to find the key factors affecting the storage of embryonic rice (Figure 7). It is mainly divided into two transcription processes, the green box for fatty acid biosynthesis and the yellow box for linoleic acid metabolism. In the biosynthesis of fatty acids, there are 11 DEGs (*ACSL*, *fadD*, *ACAA1*, *MFP2*, *ACOX1*, *FabF*, *FabG*, *FabZ*, *FabI*, *MECR*, *FATB*). Acetyl-CoA is the starting material of fatty acid synthesis, and the *FabF* gene indirectly regulates the distribution of acetyl-CoA-derived fatty acid products by the catalysis of the fatty acid chain extension. *FabF* works synergistically with other enzymes (e.g., *FabG*, *FabZ*, *FabI*) to determine the length and saturation of the fatty acid chain. At 4 °C, the activity of the *FabF* gene is inhibited, and the feedback inhibition is weak, while at 25 °C, the accumulation of fatty acids increases, and the feedback inhibition is stronger, resulting in the downregulation of *FabF* expression. The *FabG* gene encodes β-ketoacyl-ACP reductase. This enzyme is the first reductase in the fatty acid synthesis cycle, and its activity determines the amount of β-hydroxylacyl-ACP production, which in turn affects the final yield of fatty acids in subsequent reactions. The *FabZ* gene encodes β-hydroxyl-ACP dehydration, and the *FabI* gene encodes enoyl-ACP reductase, which enables the fatty acid synthesis cycle to proceed smoothly to the next stage of reaction. The activity and specificity of *FabI* enzymes have an important effect on the saturation of fatty acids. In embryo rice, changes in its activity may lead to changes in the ratio of saturated and unsaturated fatty acids. The activity of *MECR* directly affects the production of branched-chain fatty acids. If the expression of *MECR* is upregulated, the synthesis of branched-chain fatty acids increases, which may change the composition of fatty acids in the rice. The *FATB* gene encodes Acyl-ACP thioesterase B, which is responsible for the termination step of fatty acid synthesis, releasing mature fatty acids. The more fatty acids that are produced, the higher the fatty acid value (Figure 1A). Palmitic acid, as a fatty acid, is regulated by enzymes encoded by *ACSL* and *fadD* genes during the storage of embryonic rice, and its expression changes can be used as a molecular index to evaluate the storage stability of embryonic rice. It is significantly downregulated at 25 °C and significantly upregulated at 4 °C.

In the metabolism of linoleic acid, acetyl-CoA (acetyl-CoA), as a precursor, participates in the synthesis of fatty acids, and the generated linoleic acid is further combined with glycerol diester through the CDP-choline pathway to form lecithin, which constitutes the main phospholipid component of the cell membrane [55]. There were 10 DEGs involved in linoleic acid metabolism. Lecithin is metabolized by *TGL4* in three steps: α-linolenic acid, linoleate, and arachidonate. The generation of α-linolenic acid also requires the action of *LCAT3* [56]. *LOX* further breaks down polyunsaturated fatty acids (such as linoleic acid and linolenic acid) into malondialdehyde by oxidizing them, triggering a chain reaction that leads to rancidity [57], nutrient loss, and odor. It can be seen from Figure 7 that *LOX* (*LOX2S*; *LOX1_5*) is significantly upregulated at 4 °C in the late storage period, while malondialdehyde is also higher at 300 d at 4 °C. Both LTB_4_ and PGF2α indirectly promote the breakdown of fatty acids and MDA production in the embryo rice by inducing oxidative stress, resulting in elevated fatty acid values and a deterioration in quality. The three DEGs of *LTA4H*, *PTGES2*, and *CBRI* act on LTB_4_ and PGF2α, which are significantly upregulated at 4 °C. JA-COA is formed by the formation of 13 (s)-HpoTrE into 12,13-EoTre under the metabolism of *AOX*, followed by *ACX* and MFP2 [58].

JA-COA induces the expression of CAT and other enzymes, inhibits the activity of *LOX*, and reduces the rate of malondialdehyde production, so the production of malondialdehyde was slow (Figure 1B).

## 4. Conclusions

In this study, PET/AL/PE was used to package embryo rice and analyze its changes in quality during storage at two temperatures, highlighting the changes in volatile compounds and gene regulation, which provided a suitable material for the storage of embryo rice, and can effectively delay the shelf life of embryo rice, especially at 4 °C. The comprehensive comparison showed that the fatty acid value of embryo rice increased continuously, and malondialdehyde and lipase increased first and then decreased. CAT and DPPH decreased, FRAP increased, and texture, taste value, and color all decreased. When the fatty acid value of embryo rice exceeds 35 mg/100 g of KOH and the comprehensive score of the taste value is lower than 60, the consumption should be stopped immediately.

A total of 72 volatile compounds were identified during embryo rice storage. Considering the threshold of volatile compounds, the main factors affecting the storage quality of embryo rice were aldehydes. 2-Pentylfuran, naphthalene, and styrene contributed the most in the early stage. 2-Hexenal, nonanal, trans-2-nonenal, and ethanol contributed more in the later stages. Correlation analysis showed that fatty acids, malondialdehyde, lipase, and FRAP were positively correlated with aldehydes and acids, while CAT and DPPH were negatively correlated. Color has the strongest correlation with volatile compounds, followed by taste value, and finally texture. Furthermore, transcriptomic analysis showed that different temperatures affected the expression of related genes during storage. During the storage period, the expression of genes related to lipid synthesis and the oxidation pathway of *LOX*, *LTA4H*, and *CBR1* was regulated to inhibit the activity of related enzymes, thereby delaying the rate of lipid oxidation and protecting the storage quality of embryo rice. A subsequent study could improve the storage period of embryo rice by controlling the lipid synthesis and oxidation of embryo rice. The results can provide a theoretical basis for delaying the quality deterioration of embryo rice and selecting storage methods, and can provide reference value for the storage of rice with a short shelf life and easy quality deterioration.

## Figures and Tables

**Table 1:** Color changes of embryo rice at two storage temperatures.

	0 d	60 d	120 d	180 d	240 d	300 d
**25 °C**						
L*	66.52 ± 1.92 ^Aa^	65.57 ± 2.03 ^Aa^	65.49 ± 1.20 ^Aa^	63.43 ± 1.41 ^Aab^	58.75 ± 6.39 ^Ab^	58.63 ± 3.66 ^Ab^
a*	−0.11 ± 0.05 ^Aa^	−0.34 ± 0.09 ^Aa^	−0.36 ± 0.05 ^Ab^	−0.39 ± 0.04 ^Ab^	−0.39 ± 0.05 ^Ab^	−0.55 ± 0.03 ^Ac^
b*	10.55 ± 1.37 ^Ad^	12.02 ± 0.82 ^Ac^	12.62 ± 0.92 ^Abc^	13.77 ± 0.29 ^Aab^	13.94 ± 0.36 ^Aa^	14.61 ± 0.25 ^Aa^
Δ*E*	-	2.90 ± 1.54 ^Aa^	1.82 ± 1.45 ^Aab^	2.75 ± 1.28 ^Aa^	0.32 ± 0.19 ^Ab^	3.45 ± 2.80 ^Aa^
**4 °C**						
L*	66.52 ± 1.92 ^Aa^	64.95 ± 2.03 ^Aa^	64.85 ± 1.34 ^Aa^	64.06 ± 0.90 ^Aa^	57.07 ± 3.78 ^Ab^	50.63 ± 6.16 ^Bc^
a*	−0.11 ± 0.05 ^Ab^	−0.16 ± 0.03 ^Bb^	−0.28 ± 0.06 ^Bc^	−0.29 ± 0.02 ^Bc^	−0.36 ± 0.04 ^Ad^	−0.37 ± 0.02 ^Bd^
b*	10.55 ± 1.37 ^Ab^	11.71 ± 0.80 ^Aab^	12.36 ± 0.90 ^Aa^	12.75 ± 0.79 ^Ba^	12.96 ± 0.29 ^Ba^	12.87 ± 0.71 ^Ba^
Δ*E*	-	3.02 ± 1.13 ^Abc^	2.55 ± 0.79 ^Ac^	1.81 ± 0.87 ^Ac^	7.04 ± 3.91 ^Bab^	7.50 ± 5.18 ^Aa^

Note: Different superscript lowercase letters indicate significant differences (*p* < 0.05) within a row, while different superscript uppercase letters indicate significant differences (*p* < 0.05) within two temperatures.

## Data Availability

The original contributions presented in the study are included in the article/Supplementary Materials; further inquiries can be directed to the corresponding author.

## Supplementary Materials

The following supporting information can be downloaded at: https://www.mdpi.com/article/10.3390/foods14091482/s1; Figure S1: The (A) moisture content and (B) protein of embryo rice at different storage times; Table S1: E-nose28 sensor range and corresponding substances; Table S2: Analysis of relative contents of volatile compounds in embryo rice during storage. Figure S2: (A) PCA (B) VIP (C) OPLS-DA biplot of embryo rice at two temperatures; Table S3: ROAV of major volatile compounds screened by VIP.

## Abbreviations

The following abbreviations are used in this manuscript:

MDA	malondialdehyde
CAT	Catalase
DPPH	2,2-Diphenyl-1-picrylhydrazyl
FRAP	Ferric Reducing Antioxidant Power
E-Nose	Electronic Nose
HS-SPME-GC-MS	Headspace–Solid-Phase Micro-Extraction–Gas Chromatography–Mass Spectrometry
