# Supplementary material for: Integrated Volatile Compounds and Transcriptional Gene Analysis Elucidate the Deterioration Mechanism of Embryo Rice During Storage
Source: Foods. 2025 Apr 24;14(9):1482. doi: 10.3390/foods14091482 (PMC12072033; doi:10.3390/foods14091482)
Supplement: Supplementary file 1 [file foods-14-01482-s001.zip › foods-3571104-supplementary.pdf]

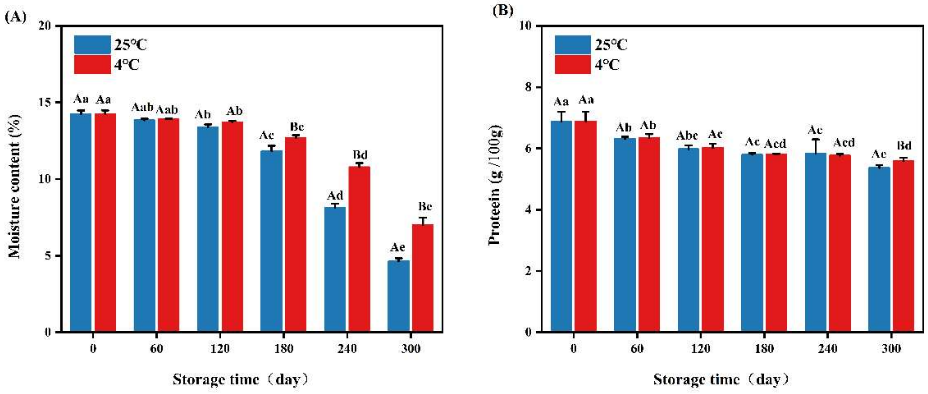

Figure S1. The (A)moisture content and(B) protein of embryo rice at different storage times. Capital letters indicate significant differences (P<0.05) at different temperatures; Lowercase letters indicate significant differences at different times (*P*<0.05).

Table S1 E-nose28 sensor range and corresponding substances

Transducers	Responsive substance	Concentration range
S1	Alkanes, fumes	200~10000ppm
S2	Alcohols, aldehydes, short-chain alkanes	1~1000ppm
S3	Ozone	10~1000ppm
S4	Sulfides, hydrogen sulfide	1~200ppm
S5	Nitrogen compounds, ammonia	5~500ppm
S6	Organic gases, phenyl ketones, alcohols aldehydes, aromatic compounds	5~500ppm
S7	Short-chain alkanes, natural gas, biogas	300~10000ppm
S8	Short-chain alkanes	300~10000ppm
S9	Sensitive to some organic solvents	0~500ppm
S10	Hydrogen	100~1000ppm
S11	Allyl sulfides	300~10000ppm
S12	Ketones, alcohols	300~10000ppm
S13	Methane	300~10000ppm
S14	Combustible gases	300~10000ppm
S15	VOC， odorless gas	1~30ppm
S16	Butane, liquefied gas	500~10000ppm
S17	Methane, natural gas	500~10000ppm
S18	Propane, Butane	500~10000ppm
S19	Short-chain alkanes, methane	100~1000ppm
S20	VOC, alcohol, methanol	1~50ppm
S21	Amines and gases with sulfur odor	1~200ppm
S22	Aliphatic hydrocarbons, alicyclic hydrocarbons, halogenated hydrocarbons	1~100ppm
S23	Alkanes, Olefins	1~30ppm
S24	Polluted air, hydrogen	30~1000ppm
S25	Methane, propane	300~10000ppm
S26	Organic acid esters and terpenes	500~10000ppm
S27	Sterols	1~30ppm
S28	Refrigerant gases	100~10000ppm

Table S2 Analysis of relative contents of volatile compounds in embryo rice during storage

NO		Compounds	RT	R.I	Temp	Relative content %					
						0day	60days	120days	180days	240days	300days
	Phenols										
1		2,4-Di-tert-butylphenol	46.74	1539	25℃	3.41	8.36	4.35	7.73	0.45	0.23
					4℃		7.84	10.68	9.90	0.30	0.21
2		2-Methoxy-4-vinylphenol	47.44	1272	25℃	10.60	9.15	5.43	9.70	1.37	2.39
					4℃		8.55	10.91	16.33	0.48	0.31
3		Guaiacol	26.07	1059	25℃	-	0.00	0.00	0.00	0.00	0.00
					4℃		0.00	0.00	0.00	0.00	0.01
	Alcohols										
4		1,2-Pentanediol	45.42	-	25℃	-	1.14	0.69	1.08	0.06	0.04
					4℃		0.00	0.00	0.00	0.00	0.00
5		Ethanol	3.09	443.2	25℃	-	0.00	0.00	0.00	58.60	51.96
					4℃		0.00	0.00	0.00	73.04	66.98
	Aldehydes										
6		Z-3-Phenylacrylaldehyde	36.58	1179	25℃	1.47	1.68	1.77	2.18	2.79	2.81
					4℃		1.70	1.88	2.42	2.58	3.32
7		2-Hexenal	24.31	820	25℃	1.65	1.88	1.97	2.32	2.68	2.88
					4℃		1.80	2.05	2.04	2.60	3.26
8		5,6-Dihydro-2H-pyran-2-carbaldehyde	44.07	-	25℃	0.00	0.47	0.51	0.83	1.27	1.35
					4℃		0.54	0.63	0.73	1.02	1.77
9		2,5-Dimethylbenzaldehyde	33.29	1154	25℃	0.00	0.98	1.26	1.35	1.78	1.88
					4℃		0.00	0.00	0.00	0.00	0.00
10		Benzaldehyde	17.81	927.2	25℃	0.00	0.00	0.00	0.00	0.65	0.77
					4℃		0.00	0.00	0.00	0.52	0.67
11		trans-2-Nonenal	18.40	1133.3	25℃	0.00	0.00	0.00	0.00	0.27	0.32
					4℃		0.00	0.00	0.00	0.17	0.29
12		Isovanillin	51.96	1468.6	25℃	0.00	1.84	2.11	2.24	2.57	2.54
					4℃		1.79	2.08	2.25	2.60	3.47
13		Decanal	17.44	1183	25℃	-	0.00	0.00	0.00	0.25	0.29
					4℃		0.00	0.00	0.00	0.00	0.00
14		trans-Cinnamaldehyde	35.74	1227	25℃	1.53	1.77	2.19	2.41	2.93	2.94
					4℃		1.60	1.70	1.73	2.36	3.04
15		Nonanal	14.42	1081	25℃	0.00	0.00	0.00	0.00	1.38	1.52
					4℃		0.00	0.00	0.00	0.00	2.01

Table S2 Analysis of relative contents of volatile compounds in embryo rice during storage

NO		Compounds	RT	R.I	Temp	Relative content %					
						0day	60days	120days	180days	240days	300days
	Ketones										
16		3-Methyl-2-cyclohexen-1-one	29.23	1039	25℃	0.00	1.01	0.37	1.43	0.18	1.88
					4℃		1.00	1.17	1.31	0.14	0.24
17		Acetone	23.00	475.3	25℃	0.40	0.40	0.44	0.52	0.57	0.82
					4℃		0.36	0.40	0.47	0.57	4.06
18		2-Heptanone	26.21	871	25℃	0.34	0.39	0.44	0.49	0.68	0.71
					4℃		0.36	0.44	0.48	0.57	0.78
19		4-Methylacetophenone	20.20	1157	25℃	-	0.00	0.00	0.00	0.31	0.18
					4℃		0.00	0.00	0.00	0.15	0.25
20		2-Oxo-2-phenylethyl formate	23.22	-	25℃	-	0.00	0.00	0.00	0.54	0.55
					4℃		0.00	0.00	0.00	0.70	0.87
	Acids										
21		Nonanoic acid	32.26	1268	25℃	0.00	0.00	0.00	0.00	0.00	0.07
					4℃		0.00	0.00	0.00	0.17	0.44
22		Octanoic acid	30.26	1154	25℃	0.00	0.00	0.00	0.00	0.00	0.30
					4℃		0.00	0.00	0.00	0.00	0.14
23		1,2,4-Benzenetricarboxylic acid, 1,2-dimethyl ester	2.32	-	25℃	0.00	0.00	0.00	0.00	5.04	3.98
					4℃		0.00	0.00	0.00	0.68	1.56
24		Acetamide	56.09	719.8	25℃	-	4.73	2.27	4.05	0.24	0.13
					4℃		2.00	2.67	2.96	0.20	0.13
25		Hydrogen azide	7.66	1213	25℃	-	0.00	0.00	0.00	0.00	3.37
					4℃		0.00	0.00	0.00	0.00	0.00
26		Formic acid	19.09	543	25℃	-	0.00	0.00	0.00	1.57	4.05
					4℃		0.00	0.00	0.00	0.75	2.28
27		Propionic acid	19.69	702	25℃	-	0.00	0.00	0.00	1.22	6.57
					4℃		0.00	0.00	0.00	0.59	2.31
	Hydrocarbons										
28		Styrene	18.71	914	25℃	5.31	6.05	3.04	1.81	0.00	0.00
					4℃		6.91	8.58	2.20	0.00	0.00
29		1,3-DI-TERT-BUTYLBENZENE	24.21	1245	25℃	9.35	0.00	21.27	0.00	0.00	0.00
					4℃		0.00	0.00	0.00	0.00	0.00
30		m-Xylene	7.02	853.2	25℃	0.00	0.00	0.00	0.00	0.70	0.00
					4℃		0.00	0.00	0.00	0.00	0.00

Table S2 Analysis of relative contents of volatile compounds in embryo rice during storage

NO	Compounds	RT	RI	Temp	Relative content %					
					0day	60days	120days	180days	240days	300days
31	Toluene	10.42	755.4	1074	0.00	0.00	0.00	0.00	0.61	0.31
				4℃		0.00	0.00	0.00	1.68	0.44
32	Dodecane	8.91	1200	25℃	0.00	0.00	0.00	0.00	0.46	0.47
				4℃		0.00	0.00	0.00	0.44	0.30
33	1,2-diethenylbenzene	24.81	1102	25℃	0.00	3.69	1.11	0.00	0.00	0.00
				4℃		2.88	0.00	3.78	0.00	0.00
34	5-Methoxybenzofuran	38.12	-	25℃	0.00	0.00	0.00	0.00	0.00	0.00
				4℃		0.00	0.00	0.35	0.00	0.00
35	Benzothiazole	38.80	1187	25℃	2.65	0.00	0.26	0.00	0.00	0.00
				4℃		0.00	0.00	0.00	0.00	0.00
36	Biphenyl	39.61	1338.4	25℃	1.74	0.00	0.00	0.00	0.00	0.00
				4℃		0.00	0.00	0.00	0.00	0.00
37	Fluorene	47.44	1552.98	25℃	0.67	0.00	0.00	0.00	0.00	0.00
				4℃		0.00	0.00	0.00	0.00	0.00
38	2-Pentylfuran	17.77	977.1	25℃	-	10.27	4.42	8.05	0.13	0.11
				4℃		10.48	12.64	12.24	0.00	0.14
39	1-methylindan	25.45	1079.7	25℃	1.48	0.00	0.00	0.00	0.00	0.00
				4℃		0.00	0.00	0.00	0.00	0.00
40	Indane	27.31	1020	25℃	0.56	0.00	0.00	0.00	0.00	0.00
				4℃		0.00	0.50	0.00	0.00	0.00
41	Methanethiol	1.94	400	25℃	0.00	0.00	0.00	0.00	0.00	0.08
				4℃		0.00	0.00	0.00	0.31	0.00
42	Naphthalene	23.21	1170	25℃	0.00	15.64	7.73	10.04	5.20	0.00
				4℃		14.35	17.83	0.00	1.83	0.00
43	METHYLENECYCLOHEXANE	23.16	731	25℃	-	17.86	5.79	0.00	0.00	0.00
				4℃		0.00	0.00	0.00	0.00	0.00
44	CADALENE	44.94	1655	25℃	1.45	0.88	0.33	0.00	0.00	0.00
				4℃		0.89	0.00	1.15	0.00	0.00
45	2,7-Dimcthylnaphthaline	39.90	-	25℃	2.23	0.00	0.00	0.00	0.00	0.00
				4℃		0.00	0.00	0.00	0.00	0.00
46	p-Cymene	21.86	1011	25℃	2.24	0.00	0.00	0.00	0.00	0.00
				4℃		0.00	0.00	0.00	0.00	0.00

Table S2 Analysis of relative contents of volatile compounds in embryo rice during storage

NO	Compounds	RT	RI	Temp	Relative content %					
					0day	60days	120days	180days	240days	300days
47	1-Methylbicyclo[3.2.1]octane	37.39	-	25℃	2.71	0.00	0.00	0.00	0.00	0.00
				4℃		0.00	0.00	0.00	0.00	0.00
48	Indole	36.77	1265	25℃	0.00	0.00	0.00	0.00	0.37	0.29
				4℃		0.00	5.14	13.82	0.40	0.30
49	Decane	3.94	1000	25℃	-	0.00	0.00	0.00	0.00	0.00
				4℃		0.00	0.00	0.00	0.27	0.19
50	Cyclopentene	16.6	560	25℃	0.86	0.78	0.85	1.43	0.10	0.05
				4℃		1.92	2.75	2.85	0.08	0.09
51	2-n-butyladamantane	30.32	1499	25℃	0.43	0.00	0.00	0.00	0.00	0.00
				4℃		0.00	0.00	0.00	0.00	0.00
52	1-tert-Butyl-3-pivaloylcyclopropene	33.21	-	25℃	8.28	1.48	0.00	0.00	0.00	0.00
				4℃		3.12	1.61	0.00	0.00	0.00
53	2-(1-Methylethyl)naphthalene	41.19	1442	25℃	0.51	0.00	0.00	0.00	0.00	0.00
				4℃		0.00	0.00	0.00	0.00	0.00
54	2,2-dimethyl-1,3-dihydroindene	29.79	-	25℃	1.00	0.00	0.00	0.00	0.00	0.00
				4℃		0.00	0.00	0.00	0.00	0.00
55	alpha.-Calacorene	37.79	1539	25℃	0.00	0.38	0.12	0.00	0.00	0.00
				4℃		0.00	0.46	0.51	0.00	0.00
56	2-Decyne	127.84	1064	25℃	0.00	0.00	1.98	2.90	0.00	0.00
				4℃		18.02	0.00	7.10	0.00	0.00
57	1-ethenyl-1h-indene	36.81	-	25℃	10.99	0.00	0.37	0.39	0.00	0.00
				4℃		0.49	0.76	0.73	0.00	0.00
58	4-Vinylphenol	48.55	1180	25℃	0.00	0.00	24.35	34.82	0.00	0.00
				4℃		0.00	0.00	0.00	0.00	0.00
59	2-Amylthiophene	25.18	1143	25℃	0.00	0.00	0.00	0.00	0.00	0.00
				4℃		1.14	1.43	1.33	0.00	0.00
60	2-Methyl-2-butene	20.27	518.6	25℃	0.00	0.00	0.00	0.00	0.00	0.00
				4℃		1.86	2.34	2.85	0.10	0.06
61	5-METHYL-3-HEXEN-2-ONE	25.35	875	25℃	0.00	0.00	0.00	0.00	0.00	0.00
				4℃		1.03	1.37	0.00	0.00	0.00
62	Cumene	19.33	914.3	25℃	1.81	0.00	0.00	0.00	0.00	0.00
				4℃		0.00	0.00	0.00	0.00	0.00

Table S2 Analysis of relative contents of volatile compounds in embryo rice during storage

NO		Compounds	RT	RI	Temp	Relative content %					
						0day	60days	120days	180days	240days	300days
63		Hexadecane	39.61	1600	1074	0.64	0.99	0.49	1.53	0.10	0.11
					4℃		0.59	0.65	0.96	0.05	0.05
64		1-Ethyl-3-methylenecyclobutane	36.51	-	25℃	0.69	0.00	0.00	0.00	0.00	0.00
					4℃		0.00	0.00	0.00	0.00	0.00
65		1,6-dimethyl-naphthalen	39.89	1442.6	25℃	1.51	0.00	0.00	0.00	0.00	0.00
					4℃		0.31	0.38	0.00	0.00	0.00
66		2,2-Dimethylbiphenyl	42.62	-	25℃	0.95	0.00	0.00	0.00	0.00	0.00
					4℃		0.00	0.00	0.00	0.00	0.00
67		cis-Calamenene	35.67	1534	25℃	0.00	4.33	0.00	0.00	0.00	0.00
					4℃		0.00	5.47	6.22	0.00	0.00
68		3-ethylstyrene	24.58	1074	25℃	8.08	0.00	2.73	0.00	0.00	0.00
					4℃		5.35	0.00	0.00	0.00	0.00
69		2-Nitropropane	11.74	631	25℃	0.00	0.00	0.00	0.00	3.32	0.01
					4℃		0.00	0.00	0.00	4.55	0.00
	Others										
70		Diphenylmethane	40.00	1380.1	25℃	0.41	0.00	0.00	0.00	0.00	0.00
					4℃		0.00	0.00	0.00	0.00	0.00
71		1-methyl-2-methylideneaziridine	30.57	-	25℃	1.46	1.39	0.56	1.53	1.58	4.03
					4℃		0.99	1.24	1.41	0.05	0.04
72		N-PHENYLPHTHALIMIDE	40.85	-	25℃	12.58	2.45	0.78	1.18	0.04	0.01
					4℃		2.15	2.24	1.88	0.05	0.02

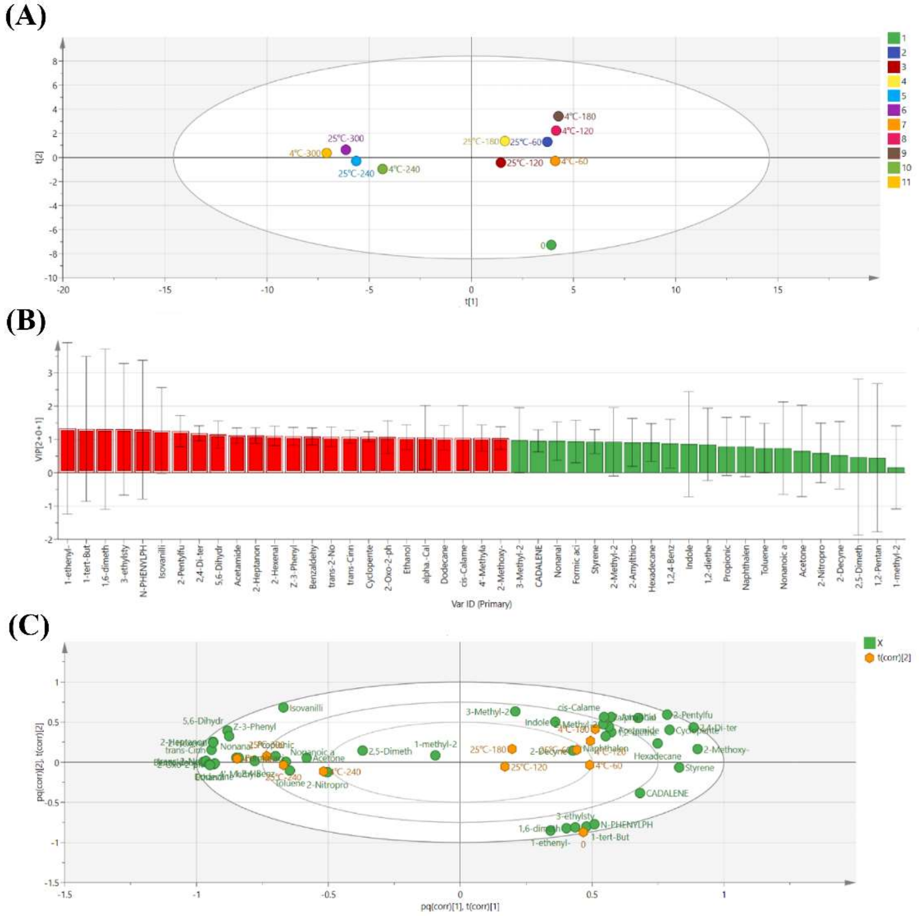

Figure S2. (A) PCA (B) VIP (C) OPLS-DA biplot of embryo rice at two temperatures.

Table S3 ROAV of major volatile compounds screened by VIP

Compounds	Odour(ng/g)	Temp	ROAV					
			0day	60days	120days	180days	240days	300days
Z-3-Phenylacrylaldehyde	0.37	25°C	0.45	0.17	0.37	0.35	6.69	7.61
		4°C		0.19	0.17	1.89	4.97	6.96
2,4-Di-tert-butylphenol	0.5	25°C	0.77	0.64	0.68	0.93	0.80	0.46
		4°C		0.66	0.73	5.72	0.42	0.32
2-Hexenal	0.03	25°C	6.23	2.38	5.13	4.65	79.10	96.09
		4°C		2.52	2.32	19.64	61.64	84.24
2-Methoxy-4-vinylphenol	0.01202	25°C	99.68	28.92	35.21	48.42	101.12	199.18
		4°C		29.87	30.85	392.68	28.25	19.89
Acetamide	140	25°C	0.00	0.00	0.00	0.00	0.00	0.00
		4°C		0.00	0.00	0.01	0.00	0.00
Acetone	0.832	25°C	0.05	0.02	0.04	0.04	0.60	0.99
		4°C		0.02	0.02	0.16	0.49	3.79
2,5-Dimethylbenzaldehyde	0.2	25°C	0.00	0.19	0.49	0.40	7.90	9.43
		4°C		0.00	0.00	0.00	0.00	0.00
trans-Cinnamaldehyde	6	25°C	0.03	0.01	0.03	0.02	0.43	0.49
		4°C		0.01	0.01	0.08	0.28	0.39
2-Pentylfuran	0.0058	25°C	0.00	67.31	59.49	83.27	19.86	18.38
		4°C		75.86	74.08	609.97	0.00	18.34
Naphthalene	0.006	25°C	0.00	99.06	100.50	100.38	769.32	0.00
		4°C		100.48	101.05	0.00	216.94	0.00
2-Nitropropane	22	25°C	0.00	0.00	0.00	0.00	0.13	0.00
		4°C		0.00	0.00	0.00	0.15	0.00
Styrene	0.0016	25°C	375.20	143.74	148.25	67.95	0.00	0.00
		4°C		181.32	182.40	397.31	0.00	0.00
Toluene	0.527	25°C	0.00	0.00	0.00	0.00	1.03	0.59
		4°C		0.00	0.00	0.00	2.27	0.65
Benzaldehyde	0.75089	25°C	0.00	0.00	0.00	0.00	0.76	1.02
		4°C		0.00	0.00	0.00	0.50	0.69
2-Heptanone	0.14	25°C	0.27	0.11	0.25	0.21	4.30	5.06
		4°C		0.11	0.11	0.99	2.88	4.32
Nonanoic acid	4.6	25°C	0.00	0.00	0.00	0.00	0.00	0.01
		4°C		0.00	0.00	0.00	0.03	0.07

Table S3 ROAV of major volatile compounds screened by VIP

Compounds	Odour(ng/g)	Temp	ROAV					
			0day	60days	120days	180days	240days	300days
Dodecane	10	25℃	0.00	0.00	0.00	0.00	0.04	0.05
		4℃		0.00	0.00	0.00	0.03	0.02
Indole	0.04	25℃	0.00	0.00	0.00	0.00	8.28	7.31
		4℃		0.00	4.36	99.85	7.11	5.75
4-Methylacetophenone	0.021	25℃	0.00	0.00	0.00	0.00	0.02	0.02
		4℃		0.00	0.00	0.01	0.01	0.02
Nonanal	0.0011	25℃	0.00	0.00	0.00	0.00	1114.45	1380.63
		4℃		0.00	0.00	0.00	0.00	1417.26
trans-2-Nonenal	0.00019	25℃	0.00	0.00	0.00	0.00	1266.08	1709.88
		4℃		0.00	0.00	0.00	644.75	1173.05
Hexadecane	0.5	25℃	0.14	0.07	0.08	0.18	0.18	0.23
		4℃		0.05	0.04	0.56	0.07	0.08
Ethanol	0.52	25℃	0.00	0.00	0.00	0.00	99.95	100.02
		4℃		0.00	0.00	0.00	100.01	99.96
Formic acid	1240	25℃	0.00	0.0	0.00	0.00	0.01	0.03
		4℃		0.00	0.00	0.00	0.00	0.02
2-Nitropropane	22	25℃	0.00	0.00	0.00	0.00	0.13	0.00
		4℃		0.00	0.00	0.00	0.15	0.00
